# Commentary: Factors affecting the knowledge levels, awareness, attitudes, and behaviors of family physicians about child abuse and neglect in Turkiye and changes in them with the training provided

**DOI:** 10.3389/fpubh.2025.1662415

**Published:** 2025-09-04

**Authors:** Schawanya Kaewpitoon Rattanapitoon, Natnapa Heebkaew Padchasuwan, Nav La, Nathkapach Kaewpitoon Rattanapitoon

**Affiliations:** ^1^FMC Medical Center of Thailand, Nakhon Ratchasima, Thailand; ^2^Faculty of Public Health, Khon Kaen University, Khon Kaen, Thailand; ^3^Faculty of Pediatrics & Medicine, International University, Phnom Penh, Cambodia

**Keywords:** child abuse, child neglect, family medicine, in-service training, primary care, health systems, medical education

Bayraktar et al. (1) provide compelling evidence on the transformative impact of in-service training on child abuse and neglect (CAaN) detection among family physicians. Their intervention achieved statistically significant improvements across knowledge domains, with effect sizes ranging from large to very large (Cohen's *d* = 1.00–3.51), markedly exceeding those reported in similar training programs for nurses and pediatric residents (2, 3). The study's rigorous pre-post design, use of validated scales, and coverage of nearly all family physicians in Nigde province strengthen its credibility and contextual relevance.

The commentary reflects a narrative literature review approach to contextualize the study. While not systematic, sources were selected based on relevance, recent publication (within the past 10 years), and citation in major databases including PubMed and Scopus. Keyword combinations such as “child abuse,” “family physicians,” “mandatory reporting,” and “training outcomes” were used in searches. Priority was given to peer-reviewed studies cited in systematic reviews, national policies, or WHO/UNICEF guidelines.

Prior research has predominantly focused on nursing students (4, 5) or hospital-based pediatricians (6), despite the central role of family physicians as first-contact providers in preventive child health systems (7). Bayraktar et al.'s study thus addresses a critical gap. Moreover, their findings raise several key reflections.

First, in terms of effectiveness, while Horstman et al. (8) emphasize the psychological burdens physicians face when reporting CAaN without adequate preparation, the present study demonstrates that structured education reduces such hesitancy by enhancing knowledge and clarifying legal responsibilities.

Second, regarding implementation gaps, their study did not evaluate whether improvements in knowledge and attitudes translated into sustained behavioral change, echoing limitations noted in similar interventions (9, 10). Cross-country reviews also reveal insufficient integration of CAaN assessments into electronic medical records to prompt routine screening (11).

To optimize CAaN detection and reporting, future research and policy should expand into the following dimensions:

1. Longitudinal impact assessment: evaluate whether knowledge gains from training lead to higher reporting rates, improved protection outcomes, and reduced re-abuse among children.
2. Interprofessional training: extend modules to include nurses, midwives, dentists, and social workers, fostering collaborative recognition and management (12).
3. Standardized interview protocols: develop culturally contextualized forensic interviewing guides for family physicians, addressing gaps highlighted in scoping reviews (11).
4. Digital decision-support tools: embed CAaN risk assessment algorithms into family health center electronic systems to prompt routine screening.
5. Legal and psychological protection policies: explore strategies to protect physicians from potential legal retaliation or psychosocial distress following mandatory reporting, as underscored in Australian and New Zealand contexts (8).
6. Ethical dimensions and international frameworks: reporting child abuse involves significant ethical tensions—particularly in balancing physician-patient confidentiality with legal duties to protect the child. These efforts should align with global standards, such as WHO's “INSPIRE” strategies and UNICEF's child protection guidelines, which emphasize frontline health worker capacity-building and institutional support mechanisms.
7. Cultural and systemic barriers in Türkiye: despite legislative frameworks, Türkiye faces barriers such as fear of retaliation, insufficient institutional support, and sociocultural stigma that deter consistent CAaN reporting. Addressing these requires legal clarity, physician support systems, and public awareness campaigns.
8. Comparative implementation models: reference successful implementation in other countries (e.g., Scandinavian models that integrate multidisciplinary CAaN teams into primary care) to highlight best practices.

## Conclusion

Bayraktar et al.'s intervention marks a critical step toward systematic CAaN prevention within Türkiye's primary care system. To sustain its impact, scaling such models nationally, evaluating their long-term outcomes, and integrating technology-driven and interprofessional frameworks will be essential. These advances may significantly enhance child safety outcomes in diverse contexts.

## References

[1] Bayraktar M Deniz S Balci E Güveli R Bircan A. Factors affecting the knowledge levels, awareness, attitudes, and behaviors of family physicians about child abuse and neglect in Turkiye and changes in them with the training provided. Front Public Health. (2025) 13:1581720. 10.3389/fpubh.2025.1581720 40612554 PMC12222197

[2] Starling SP Heisler KW Paulson JF Youmans E. Child abuse training and knowledge: a national survey of emergency medicine, family medicine, and pediatric residents and program director. Pediatrics. (2009) 123:596–602. 10.1542/peds.2008-2938 19273504

[3] Poreddi V Pashapu DR Kathyayani B Gandhi S El-Arousy W Math SB. Nursing students' knowledge of child abuse and neglect in India. Br J Nurs. (2016) 25:264–8. 10.12968/bjon.2016.25.5.264 26972999

[4] Erkut Z Gozen D Ates Besirik S. Nursing students' knowledge level on identification and risks of child abuse and neglect: a descriptive study. J Educ Res Nurs. (2021) 18:231–40. 10.5152/jern.2021.05924

[5] Kartal M Bayraktar M. The effects of undergraduate nursing education in diagnosing the symptoms of child abuse and neglect. Sakarya Med J. (2021) 11:162–9.

[6] Özer H Abakli Inci M. Knowledge and experience of pediatricians and pedodontists in identifying and managing child abuse and neglect cases. Medicine. (2024) 103:e37548. 10.1097/MD.0000000000037548 38518005 PMC10957004

[7] Evans P editor. The European Definition of General Practice/Family Medicine. Wonca Europe (2023). Available online at: https://www.woncaeurope.org/file/41f61fb9-47d5-4721-884e-603f4afa6588/WONCA_European_Definitions_2_v7.pdf (Accessed July 8, 2025).

[8] Horstman A Smith JA Bassed RB Bugeja L. The impacts on paediatricians testifying in cases of child maltreatment: a systematic scoping review. Child Abuse Negl. (2025) 163:107357. 10.1016/j.chiabu.2025.107357 40073689

[9] Calikoglu E Atila D Aktürk Z. Family physicians in an eastern Turkish city need training on child abuse and neglect. Niger J Clin Pract. (2021) 24:1766–72. 10.4103/njcp.njcp_678_20 34889783

[10] Kanat M Erkan I. Evaluation of healthcare professionals approach to child abuse and neglect. Istanbul Yeni Yuzyil Univ Yeni Yuzyil J Med Sci. (2021) 2:50–5. 10.46629/JMS.2021.41

[11] Ko H Baugerud GA Köpp U-MS Johnson MS Szyszko Hovden EA. Exploring information-gathering techniques in medical and dental interviews for child abuse and neglect: a comprehensive scoping review of existing gaps. Int J Child Maltreat: Res Policy Pract. (2024) 7:425–45. 10.1007/s42448-024-00197-w

[12] Corte-Real A Almiro PA Silva M Nunes T Abreu J Carreira C. Oral health professional intervention and child physical abuse—European legal approach. Forensic Sci Res. (2023) 8:321–7. 10.1093/fsr/owad042 38405624 PMC10894062

